# Enhancing the Activity of the DLPFC with tDCS Alters Risk Preference without Changing Interpersonal Trust

**DOI:** 10.3389/fnins.2017.00052

**Published:** 2017-02-09

**Authors:** Haoli Zheng, Siqi Wang, Wenmin Guo, Shu Chen, Jun Luo, Hang Ye, Daqiang Huang

**Affiliations:** ^1^School of Economics and Interdisciplinary Center for Social Sciences, Zhejiang UniversityHangzhou, China; ^2^School of Economics, Center for Economic Behavior and Decision-making, Neuro & Behavior EconLab, Zhejiang University of Finance and EconomicsHangzhou, China

**Keywords:** trust, risk, dorsolateral prefrontal cortex, transcranial direct current stimulation, trust game

## Abstract

Interpersonal trust plays an essential role in economic interactions and social development. Extensive behavioral experiments have examined the nature of trust, particularly the question of whether trusting decisions are connected to risk preferences or risk attitudes. Various laboratory observations have been reported regarding the difference between trust and risk, and neural imaging studies have demonstrated that the right dorsolateral prefrontal cortex (rDLPFC) is more activated when individuals decide to trust other human beings compared with individuals decide to invest in a non-social risk condition. Moreover, the rDLPFC has been found to exhibit an intimate relationship with risk preference in previous neuroscience studies. However, the causal relationship between the rDLPFC and trust has rarely been revealed. Whether modulating the excitability of the rDLPFC, which shares roles in both trust and risk decisions, alters the trust or risk preference of participants remains unknown. In the present study, we aimed to provide evidence of a direct link between the neural and behavioral results through the application of transcranial direct current stimulation (tDCS) over the rDLPFC. We found that activating the rDLPFC altered the risk preferences of our participants, whereas no such significant effect over interpersonal trust was observed. Our findings indicate that enhancing the excitability of the rDLPFC using tDCS leads to more conservative decision-makings in a risk game, and this effect is specific to non-social risk rather than social-related trust.

## Introduction

Interpersonal trust plays an essential role in economic interactions and social development (Zak and Knack, 2001; Guiso et al., 2004). Economists have debated for the nature of trust, particularly the question of whether trusting decisions are connected to risk preferences or risk attitudes. Laboratory and field experiments frequently apply the trust game to measure the interpersonal trust of participants. The trust game is occasionally called the investment game and is considered to involve risk-taking attitudes because many scientists have noted that the trust measured in the trust game might be confounded by an individual's risk preference (Karlan, 2005; Kosfeld et al., 2005; Fehr, 2009). Cook and Cooper (2003) argued that trust behavior can be clearly viewed as the trustor's risk taking choices. Because it is important to know whether trust can be predicted by risk attitude, extensive laboratory experiments have focused on the distinction between trust and risk, and various observations have been revealed. A large body of laboratory evidence has demonstrated that trust and risk are entirely different concepts with various distribution patterns in participants, whereas relatively few studies have discovered a positive relationship between risk and trust. Eckel and Wilson (2004) found no statistical relationship between behavioral risk measures and the decision to trust, and Houser et al. (2010) revealed that trust decisions are not tightly connected to a person's risk attitudes. Fetchenhauer and Dunning (2012) argued that varying the chance of repayment for trust could test the notion that trust behavior is not about risk-taking and added compelling evidence to previous results. In contrast, Schechter (2007) and Qin et al. (2011) found that behavior in the trust game is related to the actual risk-taking of participants in a non-social setting, but both of these authors measured the participants' risk preferences with dice rolling rather than measuring risk-taking behavior in a risk game that mimics the form of the trust game.

Neuroscience studies have also indicated neural differences between trust and risk-taking behaviors. In a functional magnetic resonance imaging (fMRI) experiment, McCabe et al. (2001) reported that the prefrontal regions are more active when subjects are playing with human counterparts than when playing with a computer, which represents non-social risk-taking condition. Aimone et al. (2014) provided evidence in an fMRI study that the right anterior insular cortex, as well as the medial frontal cortex and the right dorsolateral prefrontal cortex (rDLPFC), are more activated when participants play with humans in a trust game than when they play against a computer mediator in a risky decision game. Specifically, when considering trust and risk separately, the behavior of the trustor has been replicated across a large number of neuroscience studies, which indicates that the neural basis of trust lies in the activities of the prefrontal regions, striatum, amygdala, cingulate cortex, paracingulate cortex, and ventromedial prefrontal cortex (see Tzieropoulos, 2013 for a review). Transcranial direct current stimulation (tDCS) studies revealed that modulating the activity of several brain regions such as the orbitofrontal cortex, dorsolateral prefrontal cortex (DLPFC), or ventromedial prefrontal cortex may alter the participants' trustworthiness (Nihonsugi et al., 2015; Wang et al., 2016; Zheng et al., 2016). Moreover, numerous brain imaging studies have revealed the relationship between risk and the DLPFC. Because previous brain imaging studies seem to indicate that trust and risk may share the same brain region of the DLPFC, these fMRI studies have failed to demonstrate a direct causal link between the neural activities in these brain regions and trust behaviors. A causal relationship between the DLPFC and risk has been revealed in many transcranial magnetic stimulation (TMS) and tDCS studies through repeated gambling games (Knoch et al., 2006; Fecteau et al., 2007a,b; Boggio et al., 2010; Ye et al., 2015a,b). However, previous tDCS studies appling bilateral stimulation over the DLPFC failed to demonstrate the roles of the right and left DLPFC play in risk preference respectively. Crucially, the question of whether modulating the activities of the brain regions that are involved in both trust and risk, such as the rDLPFC, can influence both the interpersonal trust and risk preference of participants measured in economic interactions remains unknown. The question of whether trust is a certain kind of risk behavior has rarely been discussed in previous TMS or tDCS studies either.

In the current study, we applied midline bipolar non-balanced tDCS (see Nasseri et al., 2015; Sellaro et al., 2016 for different types of tDCS montages) over the rDLPFC to determine whether modulating the excitability of this brain area that is closely related to both trust and risk can directly influence participants' trust in a trust game as well as altering their risk preferences as measured in a risk game. After receiving tDCS stimulation (anodal, cathodal, or sham), the participants were required to complete decision-making tasks that included a trust game and a risk game. We performed the risk game following the design of Houser et al. (2010), and the form of the game was identical to the form of the trust game. The only difference between the trust and risk games was that the repayment decision was determined by human beings in the former and by a computer in the latter. Comparisons of the trust investments in the trust game between different tDCS stimulations may reveal a causal relationship between the excitability of the rDLPFC and the interpersonal trust of the participants. Furthermore, to add evidence regarding the question of whether trust is closely related to risk-taking decisions, we measured the participants' risk-taking preferences with a risk game and analyzed the correlation of trust and risk in the three stimulation groups. Moreover, if significant differences in our participants' behaviors between the different stimulations are observed in the risk game when no such influence of tDCS stimulation is discovered in the trust game, we would demonstrate that modulating the excitability of certain neural areas (such as the rDLPFC) might be specifically related to non-social risk rather than interpersonal trust.

## Experiment 1

### Materials and methods

#### Subjects

Ninty right-handed healthy subjects (mean age 21.36 years, ranging from 17 to 30 years; 46 females) with no history of neurological or psychiatric problems participated in the study for payment. All of the participants were naïve to tDCS and the trust and risk games, had normal or corrected-to-normal vision, and provided written informed consent. The protocol was approved by the Zhejiang University ethics committee. The entire experiment lasted approximately 60 min, and each participant received a payment of approximately 53.3 RMB yuan (approximately 8.076 US dollars) on average after completing their tasks. No participants reported any adverse side effects related to pain on the scalp or headaches after the experiment.

#### tDCS

In tDCS, a weak direct current is applied to the scalp via two saline-soaked surface sponge electrodes (35 cm^2^). The current is constant and delivered by a battery-driven stimulator (NeuroConn, Germany). The current was adjusted to induce cortical excitability in the target area without any physiological damage to the participant. Various configurations of the current have various effects on cortical excitability. Generally speaking, anodal stimulation enhances cortical excitability, whereas cathodal stimulation reduces the cortical excitability (Nitsche and Paulus, 2000).

**Figure 1:**
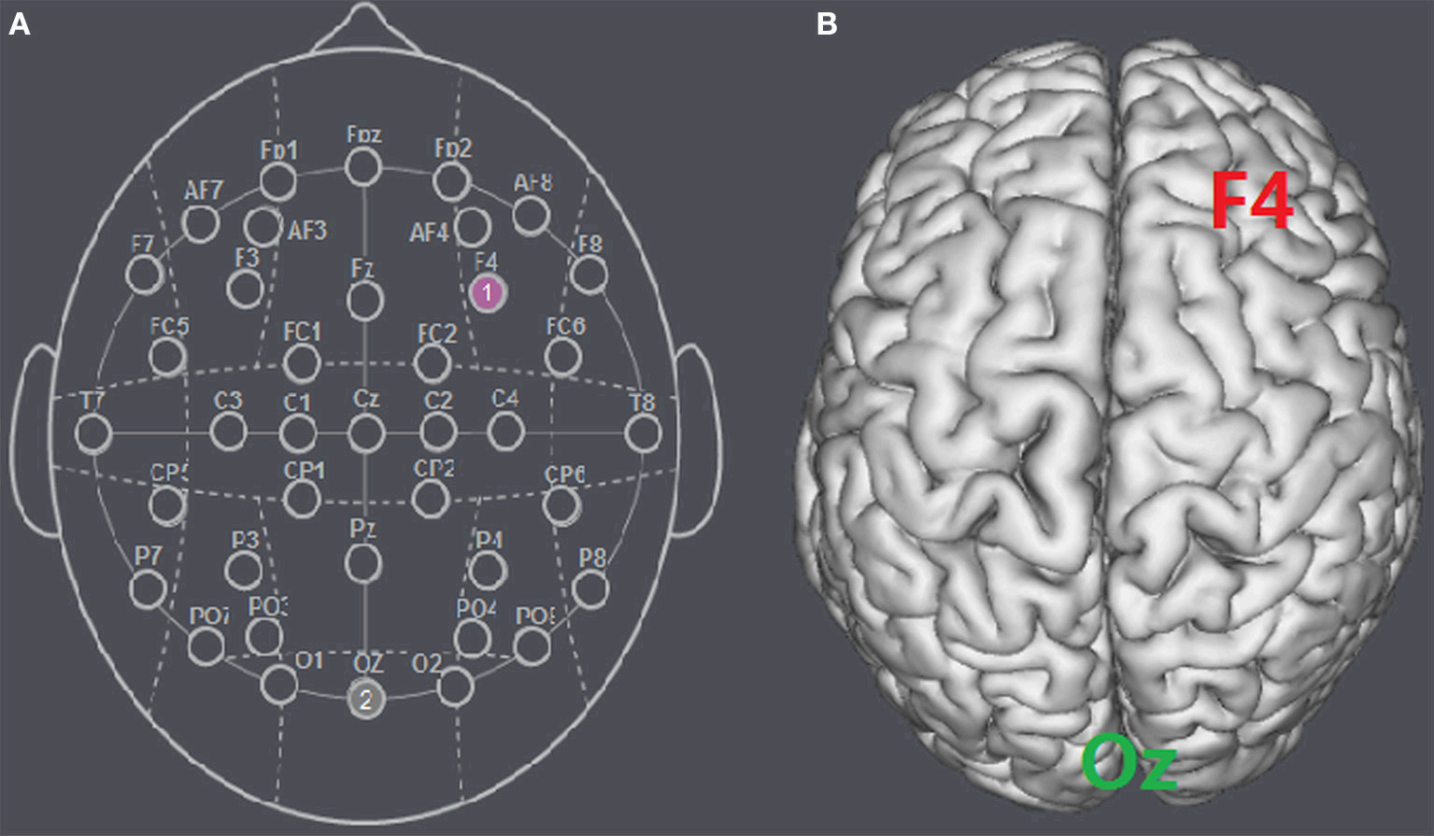
**Schematic and locations of the electrode positions. (A)** Schematic of the electrode positions F4 and Oz based on the international EEG 10-20 system. **(B)** Locations of the rDLPFC (F4) and the visual cortex (Oz) of the human brain.

**Figure 2:**
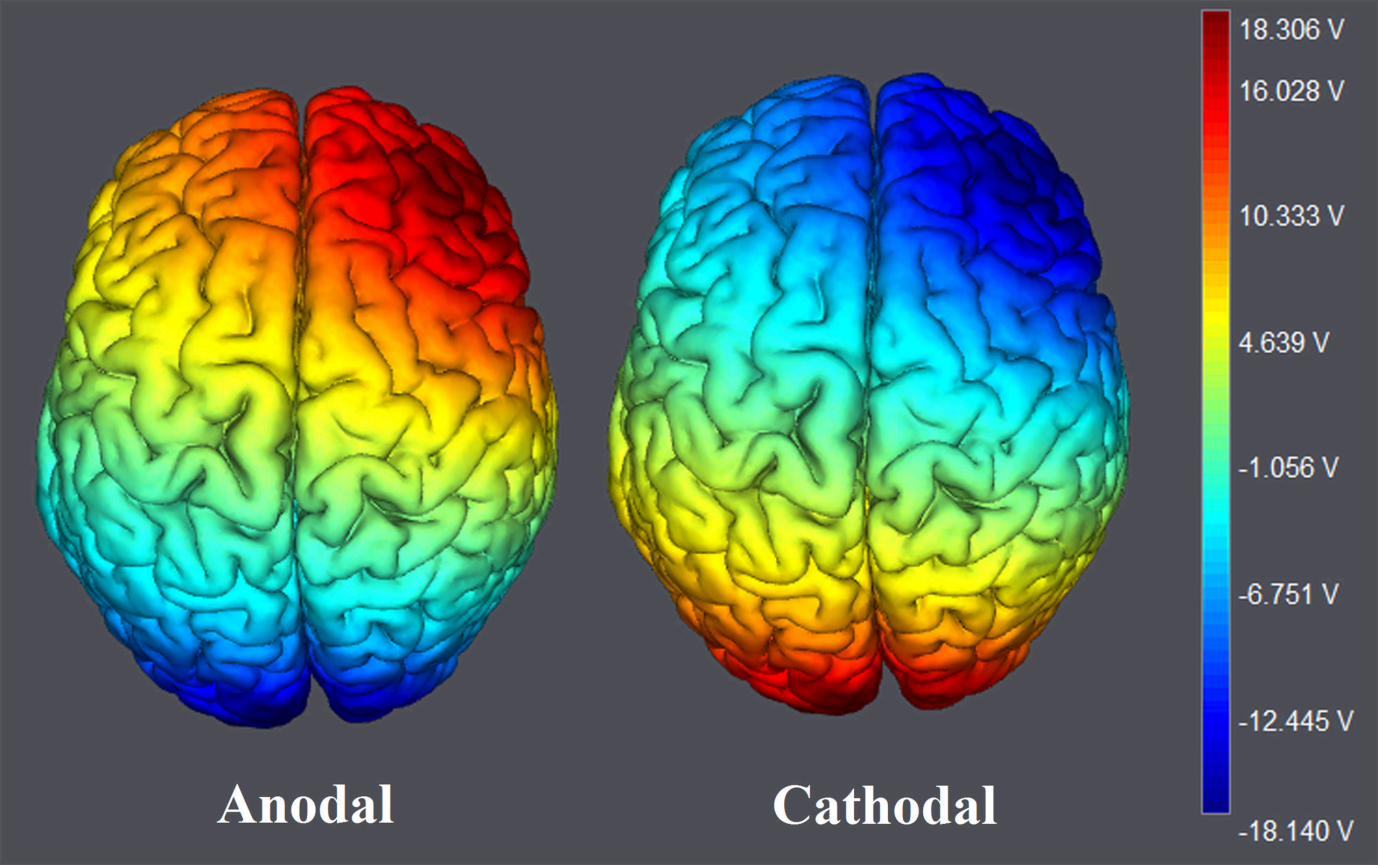
**The stimulation modes of tDCS treatments**. Electric field simulations were performed with the Neuroelectrics Instrument Controller software (version 1.3, Spain). Simulated field intensity is indicated by the color bar. The axis represents the range of input voltage from −18.140 to 18.306V.

The participants were randomly assigned to receive anodal tDCS over the rDLPFC (*n* = 30, 15 females, mean age = 21.37), cathodal tDCS over the rDLPFC (*n* = 30, 15 females, mean age = 21.43) or sham stimulation (*n* = 30, 16 females, mean age = 21.27). There was no significant age difference between the three groups [*F*_(2, 87)_ = 0.036, *p* = 0.964, η_*p*_^2^ = 0.001]. For anodal stimulation, the anodal electrode was placed over the rDLPFC, at the F4 position according to the international EEG 10/20 system, whereas the cathodal electrode was placed over the visual cortex at the Oz (Colzato et al., 2015; Nihonsugi et al., 2015). For cathodal stimulation, the cathodal electrode was placed over the F4, whereas the anodal electrode was placed over the Oz (Figures 1, 2). For sham stimulation, the procedures were the same, but the stimulation was turned off after 30 s without the participants' knowledge. The participants may have felt the initial itching, but there was actually no current for the rest of the stimulation. This method of sham stimulation has been shown to be reliable (Gandiga et al., 2006). The current was constant and of 2 mA in intensity, with a 30 s ramp up and down; the safety and efficiency of this stimulation have been demonstrated in previous studies. Before the decision making tasks, the laboratory assistant put a tDCS device on the participant's head for stimulation. After 20 min of stimulation, the tDCS device was taken off and the participant was then asked to complete two economic interaction games.

#### Task and procedure

After the participants received tDCS stimulation for 20 min (midline bipolar non-balanced stimulation, single-blinded, sham-controlled), they completed two economic decision tasks programmed by z-Tree (Fischbacher, 2007). To eliminate the sequence effect of the two tasks, we randomly assigned half of the participants performed the trust game first, and the other half performed the risk game first. Because social interaction experiments, such as the trust game, dictator game, public good game and ultimatum game, require the simultaneous interaction of a number of subjects to eliminate the participants' suspicion regarding whether they are playing with a real person, which may alter their behaviors (Frohlich et al., 2001), we managed 10 participants in the same laboratory during an experimental session. These participants were anonymous to each other and in separate cubicles.

##### Trust game

The trust game followed the classical design originally utilized by Berg et al. (1995). There are two roles in the trust game: trustor and trustee. Each role is offered a certain original endowment (for example: ten tokens). First, the trustor decides on an amount to transfer to the trustee. Next, the transferred amount is tripled and the trustee decides how much of the tripled amount to transfer back to the trustor. For example, if the amount being transferred by the trustor is X and the amount being transferred back by the trustee is Y, then the trustor will receive 10−X+Y, and the trustee will receive 10+3X−Y.

**Figure 3:**
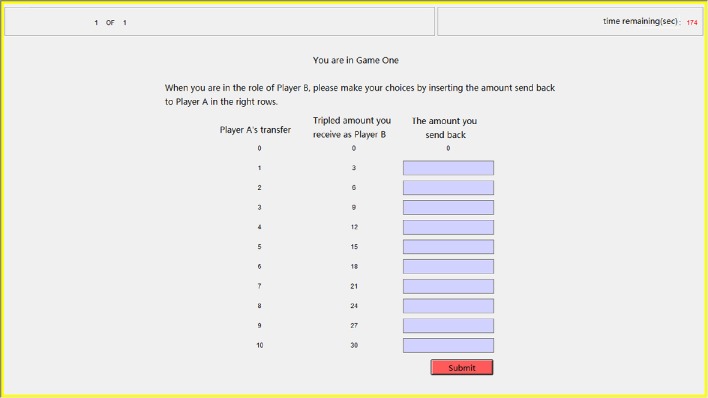
**Sample screen in the step of repayment for the trust game**. The participant playing the role of trustee had to decide the amounts sending back to the trustor in every possible condition by inputting integers in the right ten rows.

In our trust game task, after the participants passed two profit-calculating questions to ensure that they fully understood the trust game, each participant playing role A (trustor) decided how much of the original endowment (10 tokens) to transfer to the other participant playing role B (trustee). Subsequently, each participant was asked to estimate the amount send back by her partner in each possible conditions, and each correct estimation (when the difference between the estimation and the partner's choice is less than or equal to 1) was rewarded with one extra token. Our participants also made decisions when playing the role of the trustee but only the data in the role of trustor were analyzed in the current study. We used the strategy method that the trustees had to decide on a contingent action for every possible amount sent by the trustors (Figure 3), which has been proven reliable for measuring participants' trustworthiness (Brandts and Charness, 2000; Ashraf et al., 2006). Such a role reverse has also been demonstrated reliable for measuring trust and trustworthiness (Charness and Rabin, 2002; Brandts and Charness, 2011). Our participants were informed about how their decisions determined their final payments: the game was played only once with each participant randomly paired with another participant and in the final stage of the experiment, the role each participant played in this game was also randomly assigned by the computer.

##### Risk game

We applied a risk game that simplified the design of Houser et al. (2010) in our experiment. Our risk game was identical in form to the trust game. The only difference between the trust game and the risk game was that the repayment decision was determined by human beings in the former, whereas it was determined by a computer in the latter. There were also two roles in the risk game: Player A (investor) and Player B (mediator). First, Player A decided on an amount to transfer to Player B. Next, the transferred amount was tripled, and Player B decided how much of the tripled amount to transfer back to Player A. In contrast to the trust game, all of our participants played the role of Player A in the risk game, and the computer played the role of Player B. For example, if the amount transferred by Player A is X, and the amount transferred back by the Player B is Y, then Player A will receive 10−X+Y, and Player B will receive 10+3X−Y. Our participants were informed that Y would be randomly selected by the computer as a number between zero and 3X.

After our participants passed two profit-calculating questions to ensure they fully understood the game, each participant was asked how much to transfer to Player B when playing the role of Player A. The game was played only once. Our participants were also informed about how their decisions determined their final payments: in the final stage of the experiment, the payment in this experimental game was determined according to the participants' decisions and the decisions of the computer (the computer playing Player B would randomly pick an integer between 1 and 3X to transfer back to Player A. In this case, each possible integer value between 1 and 3X transferring back by the computer was equal in probability).

When the participants completed the two tasks, they were asked to complete a questionnaire before receiving their payments. The questionnaire contained questions about personal information, such as gender, age, and self-assessment of risk preference, etc. Next, our participants were shown which roles they played in each game and received their payments according to their choices and the choices of their partners or the computer. The exchange rate for game tokens and RMB yuan was 1:1, and each participant received an extra 20 RMB yuan for participation.

### Results

#### Behavioral data

In the trust game, the amount transferred (trust investment) in the role of the trustor indicated the participant's trust. The trust investment of each participant was defined as Trust (the measure of trust). The average ratio of the individual's expectations of repayment for each investment in the trust game was defined as the ExpRatio. For example, assuming that the expected repayment of the trustee was Exp1 when the trustor invested 1 token, and the expected repayment was Exp2 when 2 tokens were invested, etc., then, ExpRatio = (Exp1/1+Exp2/2+Exp3/3…+Exp10/10)/10. In the risk game, the amount transferred in the role of Player A was defined as Risk (the measure of the participant's risk preference). We also collected the data of self-asserted risk preference of each participant through questionnaire. There are 6 levels of risk preference style from 0 (very risk averse) to 5 (very risk seeking). The value of self-asserted risk preference was defined as SelfRisk.

Behavioral data were statistically evaluated using SPSS software (version 22, SPSS Inc., Chicago, IL, USA). The significance level was set at 0.05 for all analyses. We also conducted the analyses of variance (ANOVAs) within a Bayesian framework to test the evidence in favor of the null hypothesis (Rouder et al., 2012, 2016; Wetzels et al., 2012), using JASP software (version 0.8 Beta 5, University of Amsterdam, Netherland).

First, we tested whether there was any sequence effect. Although the sequences in our experiment were counterbalanced across tDCS stimulations, Trust and Risk were analyzed via ANOVA with tDCS stimulation type and sequence as between-subject factors. There was no main effect of sequence on the participants' trust [*F*_(1, 84)_ = 0.403, *p* = 0.527, η_*p*_^2^ = 0.005] or risk [*F*_(1, 84)_ = 0.021, *p* = 0.884, η_*p*_^2^ < 0.001]. The Bayesian analysis also indicated that the behaviors of participants in the trust game and the risk game were not influenced by the sequence, with evidence in favor of a model without the sequence effect as compared to a model including this factor (Trust: BF_sequence_ = 0.271; Risk: BF_sequence_ = 0.221).

Second, the observed results were similar to those of previous studies. The correlation between Risk and Trust was not significant in our anodal group participants (coefficient = −0.105, *p* = 0.582, Pearson correlation), cathodal group (coefficient = −0.060, *p* = 0.751, Pearson correlation), sham group (coefficient = −0.185, *p* = 0.328, Pearson correlation) or in the total sample including the three stimulation types (coefficient = −0.119, *p* = 0.263, Pearson correlation). The Bayesian analysis also indicated evidence in favor of the null hypothesis (BF_anodal_ = 0.262; BF_cathodal_ = 0.238; BF_sham_ = 0.358; BF_total_ = 0.244). No significant correlation between Trust and SelfRisk was observed either (Anodal: coefficient = 0.018, *p* = 0.924; Cathodal: coefficient = −0.105, *p* = 0.579; Sham: coefficient = 0.189, *p* = 0.318, Pearson correlation. BF_anodal_ = 0.228; BF_cathodal_ = 0.263; BF_sham_ = 0.365). However, the relationship between Risk and SelfRisk was significant in the total sample (coefficient = 0.226, *p* = 0.032, Pearson correlation) and in the sham group (coefficient = 0.421, *p* = 0.021, Pearson correlation). The Bayesian analysis also showed limited evidence in favor of the model with a correlation between participants' risk and their self-asserted risk preference (BF_sham_ = 5.788; BF_total_ = 2.472). Such a significant relationship between Risk and SelfRisk was not observed in the anodal group (coefficient = −0.078, *p* = 0.684, Pearson correlation; BF_anodal_ = 0.246) or in the cathodal group (coefficient = 0.204, *p* = 0.280, Pearson correlation; BF_cathodal_ = 0.397), which indicates that the anodal and cathodal stimulation may have altered the risk preference of our participants. Moreover, data of self-asserted risk preference were also analyzed by ANOVA with tDCS stimulation type as a between-subject factor. No significant effect of stimulation type was observed [*F*_(2, 87)_ = 0.732, *p* = 0.484, η_*p*_^2^ = 0.017; Anodal: mean = 2.333; Cathodal: mean = 2.567; Sham: mean = 2.600], which indicates that the tDCS stimulation has hardly any significant influence on the participant's self-asserted risk preference. There was a significant relationship between the ExpRatio and Trust in the total sample including the three stimulation types (coefficient = 0.442, *p* < 0.001, Pearson correlation). The Bayesian analysis also indicated decisive evidence in favor of the model with a correlation between participants' trust and their expectation of repayment (BF_total_ = 1534). These results obviously indicate that those who expected higher repayments exhibited more trust in the trust game. Clearly, the expectation of repayment in the risk game is 1.5 tokens for each invested token, whereas the mean ExpRatio of our participants in the trust game was 1.379 tokens for each invested token, which was significantly lower than the expectation in the risk game [*t*_(89)_ = −2.480, *p* = 0.015]. The Bayesian one sample *t*-test also showed limited evidence in favor of the hypothesis that the average ExpRatio was lower than 1.5 (BF = 4.155). The slight difference between our participants' expectations of repayment in the trust and risk games may explain the phenomenon of betrayal aversion (Bohnet and Zeckhauser, 2004; Bohnet et al., 2008) by which the individuals invested more in the risk game than in the trust game.

#### Effects of tDCS over the rDLPFC on trust

The investments of the trustors in the trust game in the anodal and cathodal tDCS over the rDLPFC and sham groups were analyzed by means of ANOVAs with tDCS stimulation type (anodal, cathodal vs. sham) as a between-subject factor. No significant effect of stimulation type was observed [*F*_(2, 87)_ = 0.103, *p* = 0.902, η_*p*_^2^ = 0.002; Bayesian ANOVA also showed that the model not including the stimulation type factor is 9.3 times more likely than the full model]. Moreover, we ran an ANCOVA with stimulation type as a between-subject factor and self-asserted risk, gender and age as covariates. No significant effect of stimulation type was observed when we controlled the factors of self-asserted risk preference, gender and age [*F*_(2, 84)_ = 0.119, *p* = 0.888, η_*p*_^2^ = 0.003]. Bayesian ANCOVA also showed that the model not including the stimulation type factor is 9.3 times more likely than the full model.

#### Effects of tDCS over the rDLPFC on risk

The investments in the risk game from the anodal and cathodal tDCS over the rDLPFC and sham groups were analyzed by means of ANOVAs with tDCS stimulation type (anodal, cathodal vs. sham) as a between-subject factor. There was a significant main effect of tDCS stimulation [*F*_(2, 87)_ = 8.236, *p* < 0.001, η_*p*_^2^ = 0.159; Bayesian ANOVA also favored the model including the stimulation type with a factor of 56.4]. *Post-hoc* analyses (Bonferroni) revealed that the risk preference in the anodal group (mean = 5.20) was significantly lower than those obtained for the sham group (mean = 7.17, *p* = 0.001) and the cathodal group (mean = 7.07, *p* = 0.003). No significant difference between the cathodal group and the sham group was observed (*p* = 0.982). We also ran an ANCOVA with stimulation type as a between-subject factor and self-asserted risk, gender and age as covariates. A significant effect of stimulation type was also observed when we controlled the factors of self-asserted risk preference, gender and age [*F*_(2, 84)_ = 8.517, *p* < 0.001, η_*p*_^2^ = 0.169]. Bayesian ANCOVA also favored the model including the stimulation type with a factor of 56.4. There was also a significant main effect of age [*F*_(1, 84)_ = 13.392, *p* < 0.001, η_*p*_^2^ = 0.138; BF_age_ = 27.2], indicating that younger participants seem to be more risk-seeking than elder ones.

**Figure 4:**
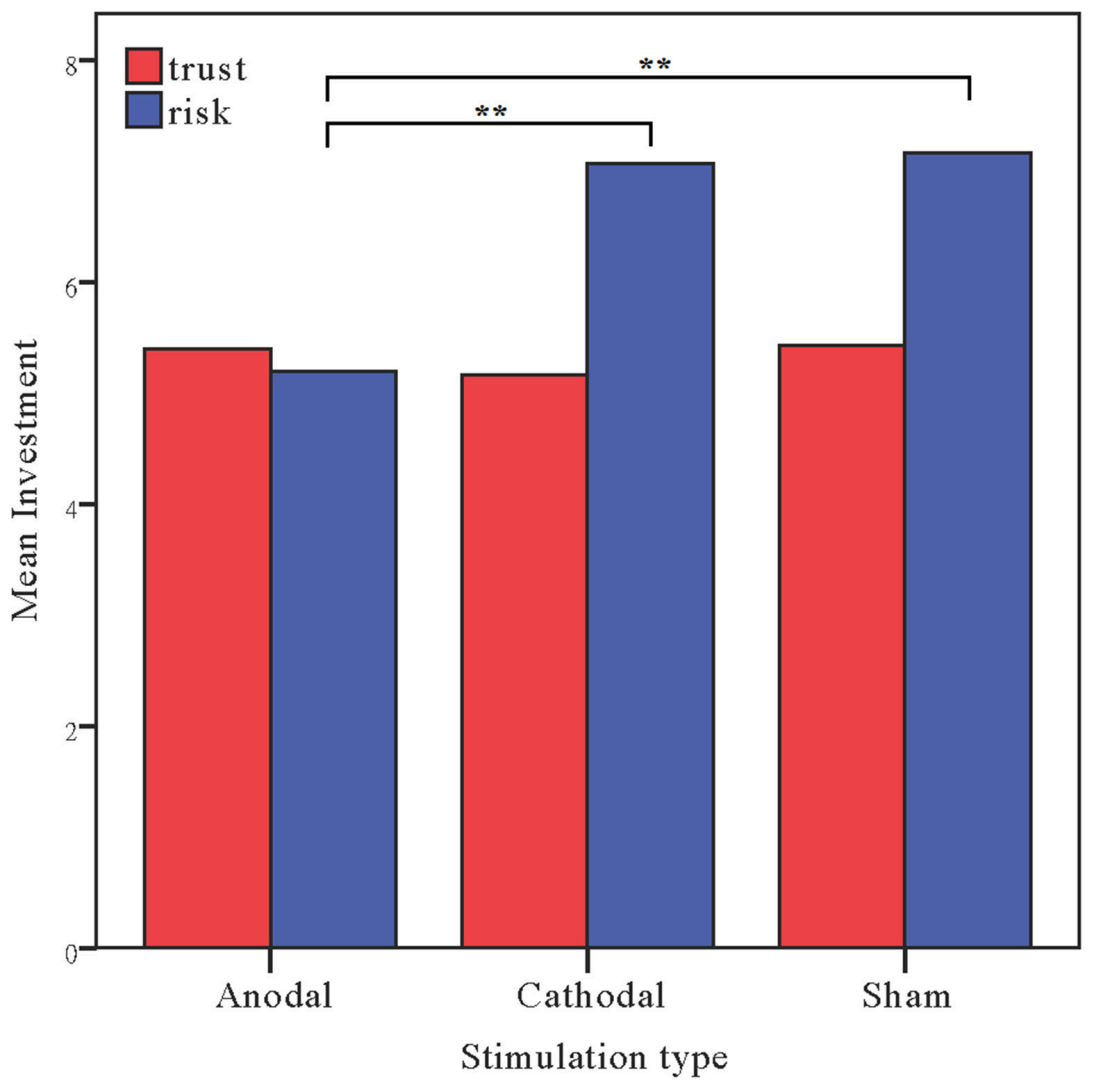
**Data of investment in the trust game and the risk game**. Mean investment in the trust game and risk game across the stimulations over the rDLPFC. The error bars indicate the 95% confidence intervals. The asterisks indicate significant differences between treatments.

Moreover, because the trust and the risk game are identical except that the repayment decision was determined by human beings in the former, whereas it was determined by computers in the latter, we ran a repeated ANOVA with task as a within-subject factor and stimulation type as a between-subject factor. There were significant main effects of task [*F*_(1, 87)_ = 9.905, *p* = 0.002, η_*p*_^2^ = 0.102; Bayesian ANOVA also favored the model including the task factor with a factor of 33.9] and tDCS stimulation type [*F*_(2, 87)_ = 3.645, *p* = 0.030, η_*p*_^2^ = 0.077; Bayesian ANOVA showed evidence in favor of the model with the stimulation type and task factors, BF = 43.5]. Crucially, significant interaction effects of task by stimulation type were observed [*F*_(2, 87)_ = 3.435, *p* = 0.037, η_*p*_^2^ = 0.073]. Bayesian repeated measures ANOVA also showed that the model with the interaction factor is 70.0 times more likely than the model not including the interaction factor (Figure 4).

### Discussion

Trusting decisions involve “the willingness to increase one's vulnerability to another who's behavior is not under one's control” (Zand, 1972), which is a type of social risk. Scholars from various disciplines have argued that risk and trust are closely related constructs (Ben-Ner and Putterman, 2001; Cook and Cooper, 2003; Schechter, 2007), whereas other social scientists have argued that risk and trust are different concepts with various distribution patterns and distinct neural bases (McCabe et al., 2001; Eckel and Wilson, 2004; Houser et al., 2010; Fetchenhauer and Dunning, 2012; Aimone et al., 2014). No significant relationship between trust and risk or between trust and self-asserted risk was observed in the anodal, cathodal, or sham groups, which adds evidence to the opinion that risk preference is uncorrelated with trust decisions, revealing that risk has little influence on participants' investments in the trust game.

Neuroimaging studies have demonstrated that the rDLPFC is of special importance for human executive function, especially in selfish impulse control (Sanfey et al., 2003; Spitzer et al., 2007; van den Bos et al., 2009). Previous neuroscience studies have also revealed that the DLPFC is activated when trustees exhibit more trustworthiness in the trust game (Chang et al., 2011; Nihonsugi et al., 2015). The rDLPFC is relatively more activated when individuals decide to trust their human counterparts than when making risky decisions while facing a computer mediator (McCabe et al., 2001; Aimone et al., 2014).

In the current study, we applied tDCS over the rDLPFC to determine the influence of the rDLPFC on trust decisions. We found that neither activating nor inhibiting the rDLPFC altered the individuals' trust investments, which indicates that changing the excitability of the rDLPFC might have no significant influence over social risk decisions such as trust. Another tDCS study also demonstrated that stimulation over the medial prefrontal cortex does not alter interpersonal trust (Colzato et al., 2015). Regarding trust behavior, many preferences, including altruism (Cox, 2004; Ashraf et al., 2006; Altmann et al., 2008), betrayal aversion (Bohnet and Zeckhauser, 2004; Bohnet et al., 2008) and ambiguity (Corcos et al., 2012) have been proven to influence individuals' interpersonal trust levels. These different preferences have various neural bases, which indicates that trust behavior involve the cooperative activities of several brain areas. Solely modulating a selfish impulse control brain region, such as the rDLPFC, may not be sufficient to alter the interpersonal trust levels of human beings.

FMRI studies have revealed that the neural activity of the rDLPFC predicts individuals' risk preferences. Using TMS, Knoch et al. (2006) discovered that disrupting the rDLPFC leads to riskier decision-making. Moreover, bilateral tDCS stimulation studies have indicated that modulating the DLPFC alters individuals' risk preferences (Ye et al., 2015a,b), and activating the rDLPFC induces more conservative choices relative to a control group (Fecteau et al., 2007a,b). Unlike the repeated gambling tasks that have been performed in previous studies, we applied a one-shot risk game that imitates the form of the trust game to test the robustness of the correlation between the rDLPFC and individuals' risk preferences in different risk-taking tasks. Consistent with previous observations, a significant effect of increasing the excitability of the rDLPFC on risky decision making was observed. The participants who received anodal stimulation exhibited more conservative behaviors than the participants in the sham group. The observations also revealed that the risk preference might be altered by tDCS stimulation for the significant correlation between risk and self-asserted risk diminished in the anodal and cathodal groups.

Although it has been revealed that anodal stimulation over rDLPFC alters risk preference of participants in Experiment 1, one may argue that the more conservative behavior shown in the anodal group was due to the cathodal stimulation over visual cortex rather than the anodal stimulation over rDLPFC. To further eliminate the possible tDCS stimulation effect over visual cortex, a control experiment was performed (Experiment 2) to rule out the influence of the visual cortex. In Experiment 2, we tested one control group (hereafter referred as “control group”) with the anodal electrode placed over rDLPFC and the cathodal electrode placed over the contralateral cheek as a reference site (Berryhill and Jones, 2012; Tseng et al., 2012; Lally et al., 2013; Mai et al., 2016). This site was chosen because it is off of the head and thus less likely to affect a response in the brain. Then, the observations including trust and risk from the control sample were compared with those from the sham group. If significant differences were also observed between the participants' risk preference from the control group and those from the sham group, we could conclude that the anodal stimulation effect over risk preference in Experiment 1 could only be due to the modulation of the specific target (rDLPFC) rather than to a cathodal stimulation effect of the visual cortex.

## Experiment 2

### Materials and methods

#### Subjects

A new sample of thirty healthy subjects (right-handed, with no history of psychiatric problem, mean age 20.90 years, ranging from 17 to 25 years; 16 females) participated in the study for payment. As in Experiment 1, all participants had normal or corrected-to-normal vision, were naïve to tDCS and the trust and risk games, and gave their written informed consent. Participants also received a written explanation of the tDCS and of any possible adverse effects without any information about the type of stimulation or the experimental hypotheses. The protocol was approved by the Zhejiang University ethics committee.

#### Apparatus, tasks, and procedure

The apparatus, tasks, and procedure were exactly the same as that in the Experiment 1 with the following exception. Since the Experiment 2 served as a control to verify that the tDCS stimulation effects observed in Experiment 1 are specific to rDLPFC, we performed one control group with the anodal electrode placed over rDLPFC, and the cathodal electrode placed over the contralateral cheek. The participants were assigned to receive anodal tDCS over rDLPFC (*n* = 30, 16 females), at the F4 position according to the international EEG 10/20 system, whereas the cathodal electrode was placed over the contralateral cheek.

### Results and discussion

Given that the participants received anodal stimulation over rDLPFC with cathodal stimulation over the cheek as a control group to verify that the more conservative behavior in anodal group observed in the risk game in Experiment 1 was due to the stimulation over rDLPFC, rather than the stimulation over visual cortex, observations measuring trust and risk preference from the control group were compared with those from the sham group. As in Experiment 1, the investments of the trustors in the trust game from the control and sham groups were analyzed with ANOVAs with tDCS stimulation type (control vs. sham) as a between-subject factor. No significant difference of the investments of the trustors between the two groups was observed [*F*_(1, 58)_ = 0.010, *p* = 0.922, η_*p*_^2^ < 0.001; Bayesian ANOVA also showed that the model not including the stimulation type factor is 3.8 times more likely than the full model]. Moreover, we ran an ANCOVA with stimulation type as a between-subject factor and self-asserted risk, gender and age as covariates. No significant effect of stimulation type was observed when we controlled these factors [*F*_(1, 55)_ = 0.135, *p* = 0.714, η_*p*_^2^ = 0.002]. Bayesian ANCOVA also showed evidence in favor of the model without the stimulation type factor (BF_stimulation_ = 0.263).

**Figure 5:**
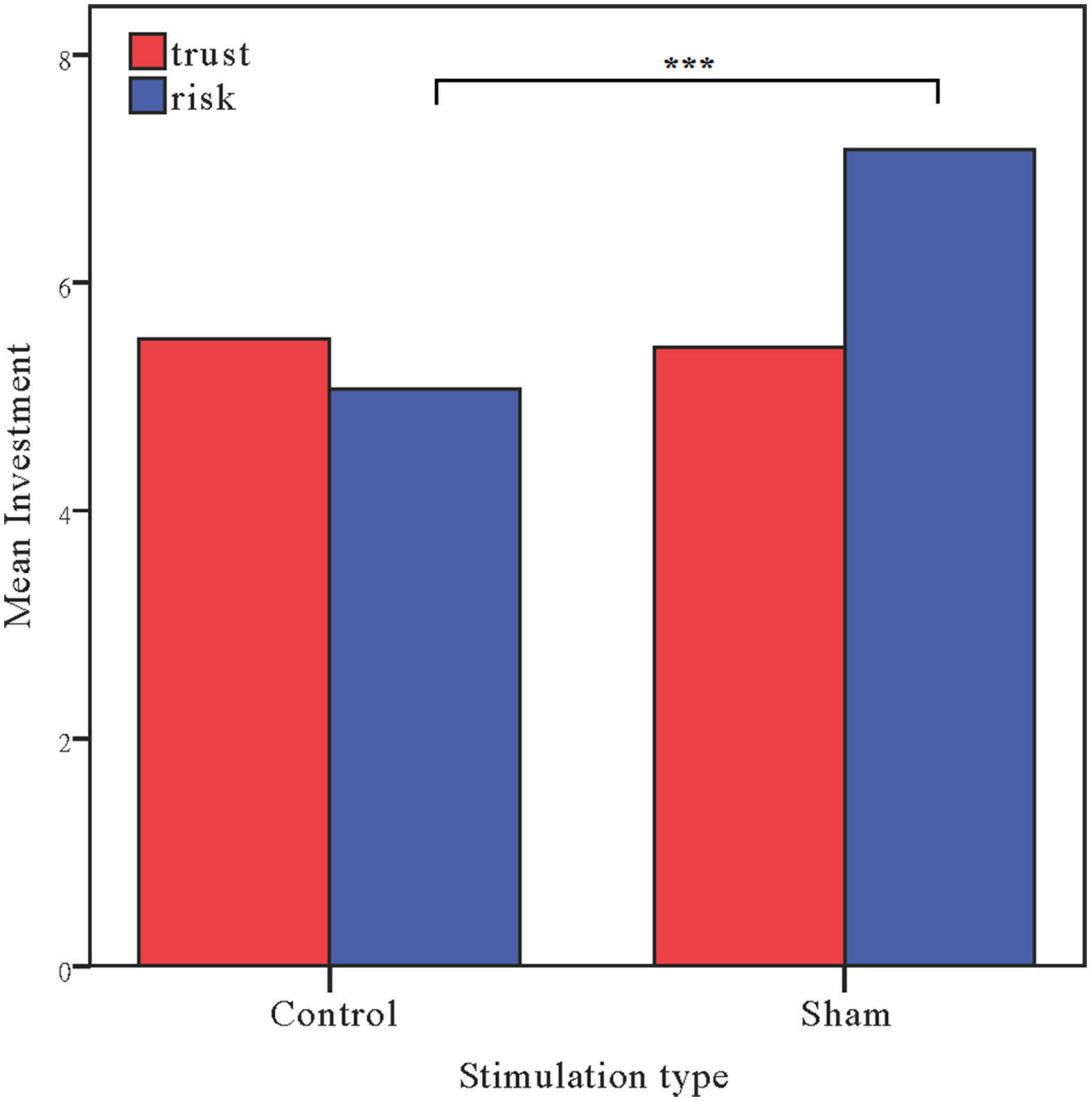
**Data of investment in the trust game and the risk game**. Mean investment in the trust game and risk game for the control and sham groups. The error bars indicate the 95% confidence intervals. The asterisks indicate significant differences between treatments.

Moreover, as in Experiment 1, the investments in the risk game from the control and sham groups were analyzed with ANOVAs with tDCS stimulation type (control vs. sham) as a between-subjects factor. There was a significant main effect of tDCS stimulation [*F*_(1, 58)_ = 15.47, *p* < 0.001, η_*p*_^2^ = 0.211; Bayesian ANOVA also favored the model including the stimulation type factor with a factor of 109.3]. The risk preference in the control group (mean = 5.07) was significantly lower than those obtained for the sham group (mean = 7.17, *p* < 0.001). We also ran an ANCOVA with stimulation type as a between subject factor and self-asserted risk, gender and age as covariates. A significant effect of stimulation type was also observed when we controlled the factors [*F*_(1, 55)_ = 14.672, *p* < 0.001, η_*p*_^2^ = 0.211]. Bayesian ANCOVA also showed decisive evidence in favor of the model including the stimulation type factor (BF_stimulation_ = 109.3). We also ran a repeated ANOVA with task as a within-subject factor and stimulation type as a between-subject factor. Significant interaction effects of task by stimulation type were also observed [*F*_(1, 58)_ = 6.450, *p* = 0.014, η_*p*_^2^ = 0.100]. Bayesian repeated measures ANOVA also showed that the model with the interaction factor is 5.576 times more likely than the model without the interaction factor (Figure 5).

The results of this experiment ruled out the possibility that the altering of behaviors in the risk game observed in anodal rDLPFC group of Experiment 1 were due to the stimulation effect over visual cortex rather than activating the excitability of rDLPFC. The results of Experiment 2 provide straightforward evidence that the anodal tDCS stimulation effect in the risk game was due to the modulation of rDLPFC.

## Conclusions

In the present study, we re-examined the question of whether trust is a type of risk-taking behavior that can be predicated by individuals' non-social risk preferences. Our observations revealed that activating the rDLPFC can induce less risky behavior in a one-shot risk game and that this causal relationship seems to be unique to non-social risk contexts because no correlation between the rDLPFC and social risk preference in the trust game was found. Our findings indicate that the trusting behavior of individuals in a trust game has multiple driving forces and results from the interaction of many neural regions. Thus, trust is much more complicated than the risk preference in human beings. We also found that interpersonal trust is not the same as risk-taking behavior, and the potential neural basis supporting cooperative decisions in the trust game remains to be exploited.

One limitation of the current study is that only one potential driving force of trust (for example, risk preference) has been discussed while many other preferences, including altruism, betrayal aversion, and ambiguity, which have been proven to influence individuals' interpersonal trust levels remain unknown. Further studies may focus on these preferences respectively to reveal the nature of trust. Another deficiency of our study is that following many tDCS studies, the current intensity of stimulation was set as 2 mA (see Sellaro et al., 2016 for a review), which does not necessarily increase efficacy of stimulation, but may also shift the direction of excitability alteration (Batsikadze et al., 2013). The distance between the target brain area and the return electrode in our study is also very large that selective stimulation of the target area may not be guaranteed. These issues should be taken into account for applications of the stimulation technique in future studies.

In summary, our findings add evidence to the argument that trust is not exactly identical to risk preference. The influence of tDCS over the rDLPFC is specific to non-social risk preference but not social risk such as trust. Activating the rDLPFC with tDCS can induce more conservative decisions in the risk game without altering interpersonal trust investments. Our results indicate that the rDLPFC is specific to the risk preferences of individuals, and no tDCS stimulation effect on the interpersonal trust of trustors was observed.

## Author contributions

HZ, SW, WG, SC, JL, HY, DH: Designed experiment; HZ, SW, WG, SC, JL, HY, DH: Performed experiment; HZ, SC: Analyzed data; HZ: Drew figures; HZ, SC, JL, DH: Wrote the manuscript; HZ, SW, WG, SC, JL, HY, DH: Revised the manuscript and HZ, SW, WG, SC, JL, HY, DH: Finally approved the version to be published.

### Conflict of interest statement

The authors declare that the research was conducted in the absence of any commercial or financial relationships that could be construed as a potential conflict of interest.
